# Brain volume in infants with metopic synostosis: Less white matter volume with an accelerated growth pattern in early life

**DOI:** 10.1111/joa.14028

**Published:** 2024-02-28

**Authors:** L. Gaillard, M. C. Tjaberinga, M. H. G. Dremmen, I. M. J. Mathijssen, H. A. Vrooman

**Affiliations:** ^1^ Department of Plastic and Reconstructive Surgery and Hand Surgery Erasmus MC—Sophia Children's Hospital, University Medical Center Rotterdam Rotterdam The Netherlands; ^2^ Department of Radiology and Nuclear Medicine Erasmus MC—Sophia Children's Hospital, University Medical Center Rotterdam Rotterdam The Netherlands

**Keywords:** brain volume, craniosynostosis, grey matter, metopic synostosis, MRI, trigonocephaly, white matter

## Abstract

Metopic synostosis patients are at risk for neurodevelopmental disorders despite a negligible risk of intracranial hypertension. To gain insight into the underlying pathophysiology of metopic synostosis and associated neurodevelopmental disorders, we aimed to investigate brain volumes of non‐syndromic metopic synostosis patients using preoperative MRI brain scans. MRI brain scans were processed with HyperDenseNet to calculate total intracranial volume (TIV), total brain volume (TBV), total grey matter volume (TGMV), total white matter volume (TWMV) and total cerebrospinal fluid volume (TCBFV). We compared global brain volumes of patients with controls corrected for age and sex using linear regression. Lobe‐specific grey matter volumes were assessed in secondary analyses. We included 45 metopic synostosis patients and 14 controls (median age at MRI 0.56 years [IQR 0.36] and 1.1 years [IQR 0.47], respectively). We found no significant differences in TIV, TBV, TGMV or TCBFV in patients compared to controls. TWMV was significantly smaller in patients (−62,233 mm^3^ [95% CI = −96,968; −27,498], Holm‐corrected *p* = 0.004), and raw data show an accelerated growth pattern of white matter in metopic synostosis patients. Grey matter volume analyses per lobe indicated increased cingulate (1378 mm^3^ [95% CI = 402; 2355]) and temporal grey matter (4747 [95% CI = 178; 9317]) volumes in patients compared to controls. To conclude, we found smaller TWMV with an accelerated white matter growth pattern in metopic synostosis patients, similar to white matter growth patterns seen in autism. TIV, TBV, TGMV and TCBFV were comparable in patients and controls. Secondary analyses suggest larger cingulate and temporal lobe volumes. These findings suggest a generalized intrinsic brain anomaly in the pathophysiology of neurodevelopmental disorders associated with metopic synostosis.

## Graphical abstract

To gain insight into the pathophysiology of metopic synostosis and its association with neurodevelopmental disorders, we studied preoperative brain volumes of non‐syndromic metopic synostosis patients compared to controls using MRI. We found smaller total white matter volume with accelerated white matter growth in metopic synostosis patients, similar to white matter growth patterns in autism. Our findings suggest a generalized intrinsic brain anomaly in the pathophysiology of metopic synostosis.

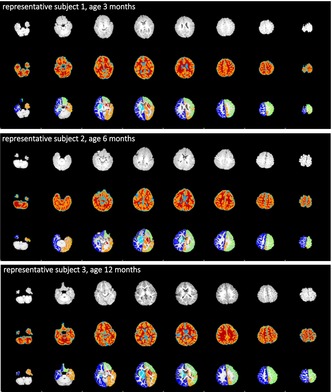

## 1 INTRODUCTION

Metopic synostosis, caused by premature fusion of the metopic suture, is the second most common form of single‐suture craniosynostosis (Cornelissen et al., 2016). The pathogenesis of metopic synostosis is not fully understood, and theories on its aetiology range from an intrinsic bone anomaly to an intrinsic brain anomaly (van der Meulen, 2012). The first theory suggests that metopic synostosis is a true malformation of the metopic suture with fusion of the frontal bones, which may lead to mechanical restriction of the brain. In contrast, the second theory suggests trigonocephaly is caused by an intrinsic brain anomaly. This theory states that malformation of the frontal lobes causes a lack of driving force required for maintaining suture patency and therefore results in premature ossification of the metopic suture (Maltese et al., 2014; Moss, 1959; Riemenschneider, 1957; van der Meulen, 2012).

We recently investigated cerebral blood flow and white matter microstructure of white matter tracts located (partially) in the frontal lobe in young unoperated patients with trigonocephaly as compared to healthy controls with arterial spin labelling and diffusion tensor imaging, respectively (de Planque, Gaillard, et al., 2022; de Planque, Petr, et al., 2022). In both studies, we found no significant difference between patients and controls. Similar studies have been conducted in syndromic craniosynostosis patients (Doerga et al., 2020; Rijken et al., 2015). In contrast to patients with metopic synostosis, cerebral blood flow was decreased in patients with syndromic craniosynostosis before surgery but normalized after surgery (Doerga et al., 2020). In addition, diffusivity parameters were increased in patients with syndromic craniosynostosis as compared to healthy controls, indicating abnormal white matter microstructure (Rijken et al., 2015). We would have expected similar results in patients with metopic synostosis if mechanical restriction had been a major pathogenic mechanism. The normal cerebral blood flow and white matter microstructure suggest there is no mechanical restriction of brain development in young unoperated metopic synostosis patients (de Planque, Gaillard, et al., 2022; de Planque, Petr, et al., 2022). However, our findings do not exclude the possibility of an intrinsic brain anomaly.

Several studies have investigated intracranial volume of patients with metopic synostosis with 3D photogrammetry or CT (Anderson et al., 2004; Maltese et al., 2014; McKee et al., 2021). Maltese et al. showed that metopic synostosis patients show a normal total intracranial volume preoperatively (Maltese et al., 2014). Although previous studies focused on intracranial volume rather than brain volume, they imply that frontal brain volume of patients with metopic synostosis may also be smaller as compared to healthy controls, which may support the intrinsic brain anomaly theory. To further investigate true brain volume in metopic synostosis, this study aimed to compare brain volumes of patients with unoperated metopic synostosis patients with healthy controls using magnetic resonance imaging (MRI) scans.

## 2 METHODS

We conducted a retrospective cohort study among patients with non‐syndromic trigonocephaly, which was approved by the Institution Research Ethics Board (IRB) at the Erasmus University Medical Center, Rotterdam, the Netherlands (MEC‐2018‐124).

### 2.1 Subjects

We included all unoperated moderate and severe non‐syndromic metopic synostosis patients for whom both T1‐ and T2‐weighted preoperative MRI brain scans were available. We excluded patients with known pathogenic variants or clinically significant chromosomal aberrations (e.g. 9p deletion syndrome, Down syndrome and Jacobsen syndrome), patients who were considered syndromic based on clinical features (i.e. presented with major additional dysmorphic features or additional congenital anomalies) and patients born prematurely. For metopic synostosis patients, data on the presence/absence of papilledema at fundoscopy and occipital frontal curve deflection were collected to assess whether patients suffered from intracranial hypertension. In addition, type of treatment and follow‐up time were recorded.

Controls were obtained from a historic hospital MRI database of children who had undergone MRI brain scans between 2010 and 2020. Patients were considered controls if cerebral and/or skull pathology was absent and if they were not born prematurely. An expert paediatric radiologist and neurosurgeon reviewed scans of controls to ensure the absence of cerebral pathology and/or skull pathology. Controls were included if they either had a T1‐weighted scan available or had both T1‐ and T2‐weighted MRI scans available and if they were younger than 2 years at the time of the MRI scan.

### 2.2 MRI acquisition

MRI data were acquired with a 1.5 Tesla unit (General Electric Healthcare, Milwaukee, Wisconsin).

Both groups underwent deep sedation or anaesthesia during the MRI procedure using sevoflurane or propofol. We included the following sequences: three‐dimensional (3D) T1‐weighted fast spoiled gradient‐recalled (FSPGR), 3D T2 CUBE, T2 PROPELLER and T2 SE dual echo, T2 FRSFE.

### 2.3 Data processing and brain volume measures

Images were resampled into a 256 × 256 × 256 matrix using a trilinear interpolation. Next, they were normalized to (0.1000). Further pre‐processing steps were performed with tools from the FMRIB Software Library (FSL) (version 6.0.0, Analysis Group, FMRIB, Oxford, UK) (Jenkinson et al., 2012). Using FLIRT, the T1‐weighted images were rigidly co‐registered to T2‐weighted images. Next, skin, bony skull and dura tissue were removed from the imaging data with BET (skull stripping). Finally, corrections were made for artefacts, translations and/or rotations resulting from head motion. Brain segmentation was performed using HyperDenseNet, a convolutional neural network developed by Dolz et al. (2019) and trained with the iSeg dataset (Sun et al., 2021; Wang et al., 2019). Scans that failed the automated segmentation were excluded. To obtain grey matter volume per lobe, a 4D infant brain volumetric atlas, developed by Chen et al., was used to label distinct cortical subregions (Chen et al., 2022). Lobe‐specific grey matter volumes were refined by combining the atlas' labelling with HyperDenseNet brain segmentations. Subregions were grouped based on FreeSurfer's lobe mapping (Table 1) (Klein & Tourville, 2012). The cingulate cortex was investigated separately, as the limbic system, of which the cingulate cortex is a part, is not a separate region in the FreeSurfer lobe mapping and the structure is located in both the frontal and parietal lobes. The cingulate cortex contained the following regions: rostral anterior, caudal anterior, posterior and isthmus. We did not aim to investigate hypotheses related to laterality for this study. Therefore, all presented volumetric measures are bilateral. Calculating lobe‐specific white matter volumes requires different techniques and is beyond the scope of this study.

**TABLE 1:** Grey matter subregions.

Lobe	Frontal	Parietal	Temporal	Occipital
Subregions	Superior frontal	Superior parietal	Superior, middle and inferior temporal	Lateral occipital
	Rostral and caudal middle frontal	Inferior parietal	Banks of the superior temporal sulcus	Lingual
	Pars opercularis, pars triangularis and pars orbitalis	Supramarginal	Fusiform	Cuneus
	Lateral and medial orbitofrontal	Postcentral	Transverse temporal	Pericalcarine
	Precentral	Precuneus	Temporal pole	
	Paracentral		Parahippocampal	
	Frontal pole		Entorhinal	

*Note*: Subregions included in each lobe are shown. Some regions cross lobular boundaries but were included in one lobe (i.e. paracentral and fusiform). Division of subregions into lobes was based on FreeSurfer's lobe mapping: https://surfer.nmr.mgh.harvard.edu/fswiki/CorticalParcellation. The cingulate was investigated separately and contained the following regions: rostral anterior, caudal anterior, posterior and isthmus.

### 2.4 Quality assessment

**FIGURE 1:**
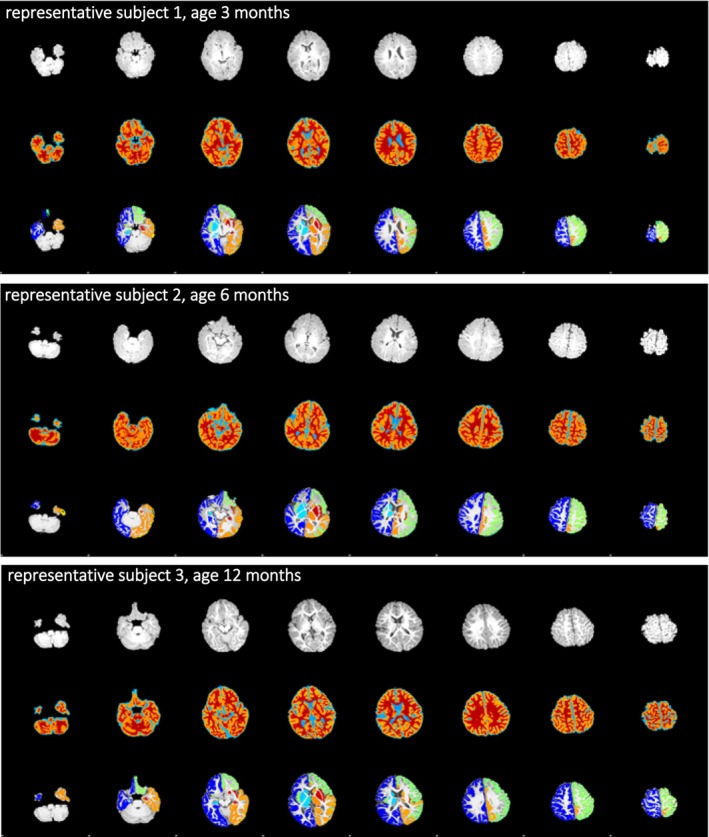
HyperDenseNet brain segmentations of three representative subjects with metopic synostosis (aged 3, 6 and 12 months, respectively). In each frame, the first row shows the plain axial MRI scan, the second row shows segmentation into grey matter, white matter and CSF and the third row shows segmentation of grey matter into subregions.

To assess the reliability and quality of our processed data, surface overlays on each MRI scan were visually inspected (representative examples are shown in Figure 1). Minimal topology errors at the internal cortical boundary were allowed, but scans with larger topology errors were excluded from our analyses. Quality assessments were discussed and agreed upon by three researchers (LG, MCT and HV).

### 2.5 Statistical analysis

We conducted statistical analyses with R version 4.1.3 (2022‐03‐10). Descriptive statistics are reported as mean with standard deviation (SD) for continuous, normally distributed data and as frequencies (%) for categorical variables. We obtained the median with interquartile range (IQR) for continuous skewed data of patient demographics. Scatterplots were created to visualize the brain volumes at different ages for patients and controls. Using Q–Q plots and residuals versus fits plots, normal distribution and homoscedasticity were assessed. Levene's test was used to evaluate equality of variances. If brain volume data were not normally distributed, data transformations were investigated. Since the increase in most brain volumes is non‐linear over time, age was log‐transformed in all linear models. When assumptions of linear regression were met, multiple linear regressions were performed. Linear regressions were used to compare absolute brain volumes of trigonocephaly patients and healthy controls. We included age and sex as confounders (Lehtola et al., 2019). Log‐transformed age, sex and the presence of metopic synostosis were entered in the multiple linear regression model to compare absolute brain volumes of metopic synostosis patients and healthy controls at the time of MRI (Yankowitz et al., 2021) For our primary analysis, total brain volume, total grey and white matter volume, cerebrospinal fluid volume and intracranial volume were assessed. In our secondary analysis, we included regional grey matter volume of the frontal, parietal, temporal and occipital lobes and cingulate cortex. For our primary analysis, a Holm correction was performed to correct for multiple testing. A Holm‐corrected *p*‐value of <0.05 was considered statistically significant.

## 3 RESULTS

MRI scans of 53 patients and 21 controls were included for image processing. After visual inspection of the processed scans, 8 patients and 7 controls were excluded due to insufficient quality of the MRI scans resulting in failed automated segmentation (1 patient and 3 controls) or topology errors (7 patients and 4 controls). In total, 59 MRI scans were included: 45 preoperative MRI scans of patients with metopic synostosis (median age at the time of MRI 0.56 years, IQR 0.36) and 14 MRI scans of controls (median age at the time of MRI 1.1, IQR 0.47) (Table 2).

**TABLE 2:** Participant characteristics.

	Patients	Controls
Participants, *n*	45	14
Median age at MRI (IQR), y	0.56 (0.36)	1.1 (0.47)
Sex		
Male, *n* (%)	33 (73)	5 (36)
Female, *n* (%)	12 (27)	9 (64)

### 3.1 Total volumes

**FIGURE 2:**
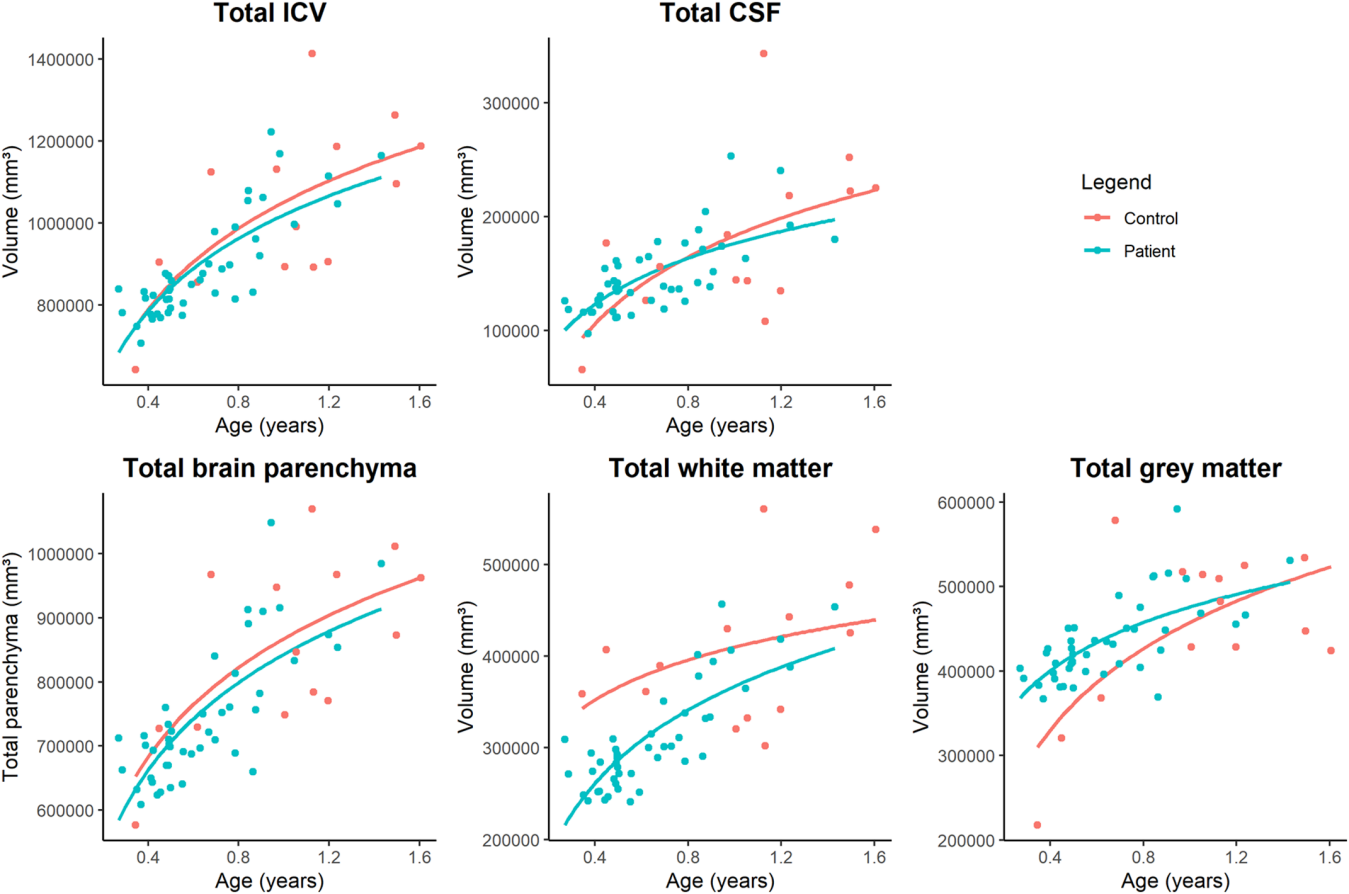
Scatter plots with a best‐fit logarithmic approximation of volume in mm^3^ as a function of age in years for metopic synostosis patients and healthy controls.

In Figure 2, scatter plots of total brain volumes with a best‐fit logarithmic approximation of volume in mm (Maltese et al., 2014) as a function of age in years for metopic synostosis patients and healthy controls are shown. We did not find a significant difference in total intracranial volume (−58,040 mm^3^ [95% CI = −125,550; 9471], Holm‐corrected *p =* 0.270), total brain volume (−51,523 mm^3^ [95% CI = −104,199; 1152], Holm‐corrected *p =* 0.220), total grey matter volume (10710 mm^3^ [95% CI = −21,770; 43,189], Holm‐corrected *p =* 1.0) or total cerebrospinal fluid volume (−6516 mm^3^ [95% CI = −32,187; 19,155], Holm‐corrected *p =* 1.0) in patients with metopic synostosis as compared to controls. However, white matter volume was significantly smaller in metopic synostosis patients (−62,233 mm^3^ [95% CI = −96,968; −27,498], Holm‐corrected *p =* 0.004). Regression coefficient estimates, standard error and 95% confidence interval (CI) for each region are shown in Table 3.

**TABLE 3:** Linear regression of total brain volumes.

	Estimate	SE	2.5% CI	97.5% CI	*p*‐value	Holm‐corrected *p‐*value
ICV						
Intercept	1,096,324	30,253	1,035,695	1,156,952	<2e‐16	
Age (years)	254,059	30,418	193,099	315,019	0.000	
Sex (male)	−73,870	27,048	−128,075	−19,665	0.008	
Metopic synostosis	−58,040	33,687	−125,550	9471	0.091	0.270
Parenchyma						
Intercept	907,440	23,605	860,134	954,746	<2e‐16	
Age (years)	189,643	23,734	142,079	237,208	0.000	
Sex (male)	−63,334	21,104	−105,627	−21,040	0.004	
Metopic synostosis	−51,523	26,285	−104,199	1152	0.055	0.220
White matter						
Intercept	425,896	15,566	394,701	457,090	<2e‐16	
Age (years)	96,834	15,651	65,469	128,199	0.000	
Sex (male)	−21,882	13,917	−49,771	6008	0.122	
Metopic synostosis	−62,233	17,333	−96,968	−27,498	0.001	0.004
Grey matter						
Intercept	481,545	14,555	452,376	510,713	<2e‐16	
Age (years)	92,809	14,634	63,481	122,137	0.000	
Sex (male)	−41,452	13,013	−67,530	−15,374	0.002	
Metopic synostosis	10,710	16,207	−21,770	43,189	0.511	1.000
CSF						
Intercept	188,883	11,504	165,829	211,937	<2e‐16	
Age (years)	64,416	11,567	41,236	87,596	0.000	
Sex (male)	−10,537	10,285	−31,148	10,075	0.310	
Metopic synostosis	−6516	12,810	−32,187	19,155	0.613	1.000

*Note*: Estimates, standard error (SE), confidence interval and *p*‐values of multiple linear regression models on the total volumes assessed with independent variables age, sex and metopic synostosis. *p*‐values were corrected with a Holm correction. A *p*‐value of <0.05 was considered statistically significant.

### 3.2 Lobe‐specific grey matter volume

In our secondary analysis, grey matter volumes were compared between patients and controls per lobe. We found larger cingulate (1378 mm^3^ [95% CI = 402; 2355]) and temporal grey matter (4747 [95% CI = 178; 9317]) volumes in patients compared to healthy controls when corrected for sex and age (Table 4). There were no significant differences in frontal, parietal or occipital grey matter volumes.

**TABLE 4:** Linear regression of regional grey matter volumes.

	Estimate	SE	2.5% CI	97.5% CI
Frontal lobe				
Intercept	9723	3343	90,523	1039
Age (years)	24,684	3362	17,948	31,421
Sex (male)	−7939	2989	−13,930	−1949
Metopic synostosis	1626	3723	−5835	9087
Temporal lobe				
Intercept	58,115	2048	54,012	62,219
Age (years)	20,623	2059	16,497	24,750
Sex (male)	−5095	1831	−8764	−1426
Metopic synostosis	4747	2280	178	9317
Parietal lobe				
Intercept	56,349	2343	51,654	61,043
Age (years)	18,521	2355	13,800	23,241
Sex (male)	−4068	2094	−8266	129
Metopic synostosis	552	2609	−4676	5780
Occipital lobe				
Intercept	45,886	2056	41,766	50,005
Age (years)	6639	2067	2497	10,782
Sex (male)	−5280	1838	−8963	−1597
Metopic synostosis	59	2289	−4528	4646
Cingulate				
Intercept	10,002	438	9124	10,879
Age (years)	2654	440	1772	3536
Sex (male)	−695	391	−1480	89
Metopic synostosis	1378	487	402	2355

*Note*: Estimates, standard error (SE) and confidence interval of multiple linear regression models on regional grey matter volumes with independent variables age, sex and metopic synostosis.

## 4 DISCUSSION

The aim of this study was to gain more insight into the associated neurodevelopmental delay in metopic synostosis by investigating brain volume preoperatively. We found significantly smaller white matter volume in patients compared to controls. In our cohort, this difference was especially large very early in life and decreased with increasing age, suggesting an accelerated white matter growth pattern in metopic synostosis. We found no significant differences in intracranial volume, total brain volume, total grey matter volume or total cerebrospinal fluid volume.

The first 2 years of life are a critical period for brain development and are likely a key period in the pathogenesis of neurodevelopmental disorders such as autism spectrum disorder (Knickmeyer et al., 2008) By the age of 2 years, the brain has reached 80%–90% of adult volume (Knickmeyer et al., 2008; Pfefferbaum et al., 1994). In the first year of life, total brain volume increases by 101%, followed by a 15% increase in the second year. This vigorous growth of the brain in the first 2 years of life is predominantly driven by grey matter growth, with hemispheric cortical volume increasing 88% in the first year and 15% in the second year. In contrast, the volume of hemispheric white matter increases by 11% in the first year and by 19% in the second year.

We demonstrated that total brain volume in young metopic synostosis patients is similar to total brain volume in healthy controls preoperatively. Our findings are in line with previous studies in both patients with metopic synostosis and other types of craniosynostosis (Aldridge et al., 2017; Hill et al., 2011; Maltese et al., 2014). A previous study reported no significant differences in total intracranial volume and total brain volume of unoperated patients with unicoronal at the age of 7–72 weeks compared to age‐matched healthy controls (Hill et al., 2011). These findings suggest that total brain volume is not restricted by a dysmorphic cranium and that compensatory skull growth is sufficient to allow for brain growth in craniosynostosis patients.

Despite similar total intracranial volume, total brain volume, grey matter volume and total cerebrospinal fluid volume, patients with metopic synostosis had significantly smaller white matter volume. We observed a more pronounced difference in white matter volume in the first months of life, which appears to correct towards normal levels at the age of 2 years. These findings demonstrate that white matter volume is smaller very early in life but undergoes accelerated growth throughout the first 2 years.

Metopic synostosis has been associated with various neurodevelopmental pathologies including suboptimal general cognition, motor function, verbal and visuospatial abilities, and autistic characteristics and conduct disorders (Edwards‐Bailey et al., 2023; Gabrick et al., 2020; Mathijssen & Working Group Guideline C, 2021; Osborn et al., 2019, 2021; van der Vlugt et al., 2012). Directly extrapolating the results from our study to child development later in life is not possible. However, it is remarkable that our findings in young patients with metopic synostosis are similar to infants with autism spectrum disorder, who appear to have accelerated white matter growth in the first 2 years of life and suboptimal white matter development later in childhood (Cardenas‐de‐la‐Parra et al., 2021; Courchesne, 2004; Courchesne et al., 2007). A more gradual prolonged development is thought to result in increased cortical volumes and may allow for improved fine‐tuning of neural networks through greater environmental interaction (Deoni et al., 2016). Taken together, these studies and our results imply that accelerated white matter growth contributes to suboptimal neurocognitive outcomes in patients with metopic synostosis.

In contrast to total white matter volume, we did not find a significant difference in total grey matter volume in patients with metopic synostosis compared to controls. Our secondary analysis suggests larger temporal grey matter and cingulate grey matter volumes in metopic synostosis patients, with raw data suggesting that grey matter normalizes towards the age of 2 years. This normalization towards the age of 2 years was also observed for white matter. Enlargement of the inferior middle and superior temporal cortex and the posterior cingulate cortex has been associated with autism spectrum disorder and autistic cognitive style in previous studies in patients aged 5–25 years (Kobayashi et al., 2020; Yankowitz et al., 2021). However, decreased anterior cingulate cortex volume has also been reported in conditions such as autism spectrum disorder and ADHD, as well as various psychiatric conditions. This complicates the interpretation of total cingulate volume (Greimel et al., 2013; Mirza et al., 2004; Vogt, 2019). We did not have sufficient power to fully assess regional grey matter differences. To investigate grey matter further, future studies should combine volume, cortical thickness, gyrification and surface area analyses in studies involving larger sample sizes to further assess the role of abnormal brain development in the pathophysiology of neurodevelopmental disorders associated with metopic synostosis.

A theory on the relationship between metopic synostosis and altered neurodevelopment is that metopic synostosis is caused by an intrinsic brain anomaly of the frontal lobes, causing a lack of driving force required to maintain suture patency, resulting in premature ossification of the metopic suture and suboptimal neurodevelopment (Moss, 1959; Riemenschneider, 1957; van der Meulen, 2012). Although our sample size is limited, our results indicate a generalized intrinsic brain anomaly, rather than an isolated anomaly of the frontal lobes. This is supported by the fact that total white matter volume was significantly smaller in metopic synostosis patients despite sufficient intracranial and total brain volume, and our secondary analysis which shows abnormal cingulate grey matter and temporal grey matter volume. As the patient population in our current study was young at the time of inclusion, detailed information on neurocognitive outcome measures was not available. The hypothesis of an intrinsic brain anomaly as the underlying cause of metopic synostosis is supported by genetic studies, which have demonstrated that there is an overlap in pathogenic variants between metopic synostosis patients and patients with neurodevelopmental disorders (Calpena et al., 2019; Reijnders et al., 2018). In addition, pathogenic variants in SMAD6, the most common monogenic cause of metopic synostosis, are correlated with suboptimal neurodevelopment in non‐syndromic craniosynostosis (Calpena et al., 2020; Di Rocco et al., 2023; Wu et al., 2020). With the exception of some monogenic causes, including SMAD6, the aetiology of metopic synostosis is still poorly understood, owing to a likely multifactorial aetiology, involving a complex interplay of environmental and (epi)genetic factors (Tonne et al., 2020; Wilkie et al., 2017). Future studies should further investigate brain and specifically grey matter development, including gyrification, surface area, cortical thickness, regional volumes and cortical microstructures during childhood combined with neurocognitive testing and genetic studies to gain more insight into the neurobiological basis of neurocognitive disorders associated with metopic synostosis.

Our study has some limitations. First, accurate automatic brain segmentation is difficult in young children as tissue contrast between white and grey matter is suboptimal in this population. To ensure the reliability and quality of our data, successful processing was confirmed through manual inspection of surface overlays on each MRI scan. However, minimal topology errors at the internal cortical boundary were allowed as the small error margin was consistent across all scans and automated methods are more reproducible compared to manual segmentation despite these minor errors. Second, neurodevelopmental disorders often become apparent later in life and subtle neurodevelopment and neurocognitive functioning cannot be reliably assessed in very young patients. Therefore, we were unable to relate our findings to neurocognitive outcomes in our population. We recommend future studies on brain development incorporate neurocognitive outcomes in childhood in their study design. Finally, there was a significant difference in sex and age between patients and controls, which may have affected our effect estimates. Sex and age are known to affect brain volume. Males have been described to have larger intracranial volume, total brain volume, more cortical grey matter and more cortical white matter (Gilmore et al., 2007; Knickmeyer et al., 2008; Lehtola et al., 2019). Previous research implies growth patterns of cortical and subcortical structures in males and females are highly similar (with the exception of ventricular volume) (Knickmeyer et al., 2008). However, Holland et al. found slightly faster whole brain growth in the first 90 days of male infants (66%) compared to females (63%) (Holland et al., 2014). To adjust our effect estimates for these confounders, sex and age were included in our regression analyses as covariates. However, we may have slightly overestimated the observed associations as some residual confounding may still be present.

## 5 CONCLUSION

We found decreased total white matter volume in patients with metopic synostosis as compared to controls. Raw data suggest an accelerated white matter growth pattern in metopic synostosis, similar to white matter growth patterns seen in autism. In contrast, total intracranial volume, total brain volume, cerebrospinal fluid volume and total grey matter volume were comparable in patients and controls. Secondary analyses suggest larger cingulate and temporal lobe volumes. Taken together, these findings suggest a generalized intrinsic brain anomaly in the pathophysiology of metopic synostosis.

## AUTHOR CONTRIBUTIONS

LG contributed to study design, performed data acquisition and statistical analyses and drafted the manuscript. MCT contributed to additional data acquisition, statistical analyses and drafting of the manuscript. MD and IM contributed to the study design and critical revision of the manuscript. HV performed data analysis and contributed to the study design and critical revision of the manuscript.

## CONFLICT OF INTEREST STATEMENT

The authors have no conflicts of interest or financial disclosures to report.

## Data Availability

The original contributions presented in the study are included in the article/Supplementary Material; further inquiries can be directed to the corresponding author.
